# Genetic Modification Approaches for Parasporins *Bacillus thuringiensis* Proteins with Anticancer Activity

**DOI:** 10.3390/molecules26247476

**Published:** 2021-12-10

**Authors:** Miguel O. Suárez-Barrera, Lydia Visser, Paola Rondón-Villarreal, Diego F. Herrera-Pineda, Juan S. Alarcón-Aldana, Anke Van den Berg, Jahir Orozco, Efraín H. Pinzón-Reyes, Ernesto Moreno, Nohora J. Rueda-Forero

**Affiliations:** 1 Facultad de Ciencias Médicas y de la Salud, Instituto de Investigación Masira, Universidad de Santander, Bucaramanga 680003, Colombia; miguel.suarez@udes.edu.co (M.O.S.-B.); diseno.molecular@udes.edu.co (P.R.-V.); biomol.investigacion@udes.edu.co (D.F.H.-P.); biomol.investigacion_1@udes.edu.co (J.S.A.-A.); ehpinzon@udes.edu.co (E.H.P.-R.); 2 Corporación de Ciencias Básicas Biomédicas, Universidad de Antioquia, Medellín 050010, Colombia; 3 Department of Pathology and Medical Biology, University Medical Center Groningen, University of Groningen, 9700 RB Groningen, The Netherlands; l.visser@umcg.nl (L.V.); a.van.den.berg01@umcg.nl (A.V.d.B.); 4 MaxPlanck Tandem Group in Nanobioengieneering, Institute of Chemistry, Faculty of Natural and Exact Sciences, University of Antioquia, Medellin 050010, Colombia; grupotandem.nanobioe@udea.edu.co; 5 Facultad de Ciencias Básicas, Universidad de Medellín, Medellín 050030, Colombia; emoreno@udemedellin.edu.co

**Keywords:** parasporins, Cry toxins, cancer cells, genetic improvement

## Abstract

*Bacillus thuringiensis* (*Bt*) is a bacterium capable of producing Cry toxins, which are recognized for their bio-controlling actions against insects. However, a few *Bt* strains encode proteins lacking insecticidal activity but showing cytotoxic activity against different cancer cell lines and low or no cytotoxicity toward normal human cells. A subset of Cry anticancer proteins, termed parasporins (PSs), has recently arisen as a potential alternative for cancer treatment. However, the molecular receptors that allow the binding of PSs to cells and their cytotoxic mechanisms of action have not been well established. Nonetheless, their selective cytotoxic activity against different types of cancer cell lines places PSs as a promising alternative treatment modality. In this review, we provide an overview of the classification, structures, mechanisms of action, and insights obtained from genetic modification approaches for PS proteins.

## 1. Background

*Bacillus thuringiensis* (*Bt*) is an endospore-forming aerobic bacterium with a high capacity to resist elevated temperatures and desiccation conditions, characterized by producing parasporal toxins [1]. *Bt* was first identified in 1901 by Shigetane Ishiwata, who reported that this microorganism had an infective capacity toward *Bombyx mori*. This plague caused severe damage to the silk industry in Japan [2]. At that time, the author termed the bacteria *Bacillus sotto*. A decade later, Berliner isolated a Gram-positive bacterium in *Ephesitia kuehniella* larvae in the state of Thuringia (Germany). Ignoring the identification given by Ishiwata, Berliner designated the bacterium as *Bacillus thuringiensis*, and this nomenclature has remained ever since [3].

*Bt* toxins, such as the Cry and Cytolytic (Cyt) proteins, are present in crystal form, with toxic effects in several pest vectors. A subset of the Cry proteins present in the crystals are parasporins (PSs), proteins with cytotoxic activity in human cancer cell lines [4]. The wide spectrum of the potential applications of PSs in biotechnological and biological medicine research has made *Bt* one of the most essential microorganisms used as a bio-controller and, more recently, as a producer of non-insecticidal parasporal proteins [5].

Previous studies on proteins produced by *Bt* have predominantly focused on quantifying their insecticidal potential, disregarding their possible uses in other fields of biotechnology and health. However, in recent years, new properties, such as cytotoxic activity against cancer cells, antiprotozoal activity, and lectin function, have been described for crystals extracted from various *Bt* strains [1,6,7]. Among these functionalities, the anticancer activity is of particular relevance, being the focus of this review.

## 2. Overview of the Classification and Structure of Parasporins Found in *Bacillus thuringiensis* Strains

In the search for new therapeutic agents to treat cancer, bacterial proteins have become a focus of attention in the last two decades. In 1999, Mizuki et al. conducted a bioprospecting study using around 1700 *Bt* isolates by selecting 42 candidates with no hemolytic and cytotoxic activity to test their activity against MOLT-4 cells (human lymphoblastic leukemia) [6,7]. Prasad, Seki, and their teams described the existence of a 13-kDa parasporal toxin from a *Bt* strain with dual activity, i.e., insecticidal action against Bombyx mori and antitumor activity in colon and blood cancer cell lines [1,8]. This new promising protein, termed a parasporin, is among the *Bt* proteins with biological activities that potentially allow for medical applications.

**Figure 1:**
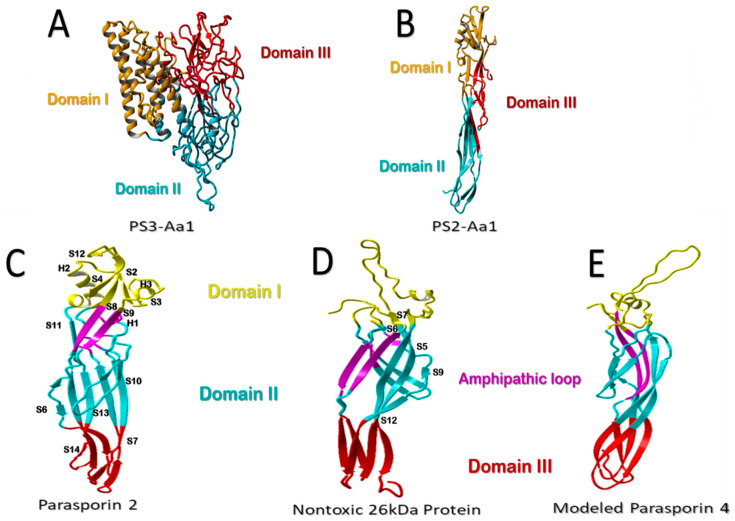
Structural comparison of parasporins. (**A**) Structural model of higher-molecular-weight PS3Aa1 with its three domains. (**B**) Low-molecular-weight PS2Aa1 structural model. (**C**–**E**) Structural comparison between parasporin-2, the 26-kDa nontoxic protein, and aerolysin-like β-PFT. Membrane-binding-related domain I is colored yellow. The membrane-insertion and pore-formation regions are colored blue (domain II) and red (Domain III). It is suggested that the purple amphipathic β-hairpin is necessary for pore formation (**C**–**E**). Parasporin 4 (PS4) was modeled using the 26-kDa nontoxic protein as an adapted template from Xu et al. [9], modified by the authors.

The Parasporin Classification and Nomenclature Committee defined the term “parasporins” in 2006 as “parasporal proteins of *Bt* and related bacteria that are non-hemolytic but are preferentially able to kill cancer cells” [9]. To date, six parasporin families (PS1–PS6) including 19 PSs produced by at least 11 *Bt* strains have been identified mainly in four countries (Japan, Vietnam, India, and Canada) (Table 1) [10,11]. PSs are divided into those of higher molecular mass (PS1, PS3, and PS6), approximately 80 kDa, which are processed into active 60 kDa molecules, and those with lower molecular mass (PS2, PS4, and PS5), originated from precursors of 33 to 37 kDa, which are processed by proteolytic cleavage to 30 kDa molecules. When a proteolytic cleavage is made by a serine protease, such as Proteinase K, at the C- terminal and N- terminal residues of the precursor, the toxin of 60 kDa and 30 kDa is active with cytocidal activity [9,10,12]. All the PS family members are characterized by a conserved structure consisting of three domains (Figure 1A,B). A more detailed structural and sequence analysis is presented by Xu et al. [9].

There are six families of parasporins described so far, from PS1 to PS6. Table 1 summarizes the families and its representative protein. Further information can be found at http://parasporin.fitc.pref.fukuoka.jp/list.html (last accessed on 5 December 2021) [20].

PS1Aa1, also known as Cry31Aa1, contains 723 amino acid residues yielding a molecular weight of 81 kDa. The crystal structure of the activated PS1Aa1 form was determined at a 1.76-Å resolution [5], revealing the typical three-domain structure established for Cry toxins. Domains I, II, and III have chain folds that make each unique, i.e., showing a set of seven α-helices, a β prism, and a lectin-like β sandwich, respectively. Cleavage of PSAa1 in the exposed loop connecting the third and fourth α-helices of domain I results in two polypeptides of 15 kDa and 56 kDa [5]. PS1 exerts strong cytotoxic effects against cell lines such as HeLa [21], HL-60 [10], and MOLT-4 [22], and a moderate effect toward Sawano, Caco-2, and Jurkat [9] cells. The IC50 values for each of the tested cell lines are summarized in Table 1.

According to Ito et al., PS2Aa1 (Cry46Aa1) and PS2Aa2 (Cry46Aa2) are 338-amino-acid polypeptides with a deduced molecular weight of 37 kDa [21]. Unlike PS1Aa1, PS2Aa1 lacks the conserved blocks found in Cry proteins. The structure of PS2 shows homology with the aerolysin of *A. hydrophila* and the alpha-toxin of *C. perfringens* [13,23], which, similarly to the PS1 and Cry insecticides, has three domains. The processing of PS2Aa1 from a 37-kDa precursor protein results in an active 30-kDa toxin [21]. In its active form, which is formed after treatment with proteinase K [24], PS2 is toxic to HepG2, Caco-2, MOLT-4, Jurkat, and HL-60 cell lines (Table 1) [14,18] but does not present any toxicity to normal cells [25].

PS3Aa1 (Cry41Aa1) consists of 825 amino-acid residues with a deduced molecular weight of 93.68 kDa and low sequence similarity with insecticidal Cry proteins. However, it contains three domains with five conserved repetitive blocks, a typical structure for PS proteins [14]. PS3Aa1 requires proteolytic digestion at its N- and C-termini for activation, thus converting the 81-kDa precursor protein into an active 64-kDa protein, with cytotoxic effects on various cancer cell lines, such as HL-60 and HepG2 (Table 1) [14].

PS4Aa1 (Cry45Aa1) is a β-pore-forming aerolysin-type protein comprising 275 amino acids, with a predicted molecular weight of 30.07 kDa [14]. No repetitive sequence blocks, as observed in PS1Aa1 and PS3Aa1, have been reported in this protein. PS4Aa1 has three domains, whose structures do not resemble those of Cry proteins [14]. The 31-kDa protoxin is activated by cleavage in its C-terminal domain by pepsins in acidic conditions, resulting in a fully active 27-kDa toxin [26]. The active protein exhibits cytotoxic activity against human cancer cell lines, e.g., Caco-2, Sawano, MOLT-4, TCS (human cervical cancer), and HL60 cells (Table 1) [9,14,17].

PS5 and PS6 are the most scarcely studied parasporins. PS5 has been isolated from *Bt* strain A1100. Sequence analysis has revealed that PS5Aa1 (Cry64Aa) is an epsilon protein that acts synergistically with the drug methotrexate [18]. Its gene sequence is 918 bp in length, encoding a 305-amino-acid polypeptide with an expected molecular weight of 38 kDa. Its C-terminus is cleaved by proteinase K, producing an active 30-kDa protein [18]. PS6Aa1 (Cry63Aa) has been isolated from the *Bt* M109 and CP84 strains [19]. Sequence analysis suggests a protein of three domains, closely related to Cry insecticides, with homology with and similarity to Cry2 of 21.9 and 56.4, respectively [19]. As for cytotoxic activity, PS5 has been shown to be active against MOLT-4, Caco-2, HepG2, TCS, HeLa, and Sawano cells, while PS6 shows weak cytotoxicity toward HepG2 cells (Table 1).

Like Cry proteins, Parasporins share structural and functional features with pore-forming toxins (PFTs), considering that its cytotoxic activity is due to the pore formation in the cell membrane [9]. There are two larger groups of PFTs, Alpha-PFTs (α-PFTs) and Beta-PFTs (β-PFTs), based on how the secondary structure of their membrane-spanning elements are composed of α-helices and β-Barrels, respectively. Within the β-PFTs, the aerolysin family includes several parasporins [27].

### β-Type-Like Pore-Forming Parasporins

The nontoxic protein of the A1470 *Bt* strain and the toxic PS2Aa1 have similar structures, especially in domain II. Both molecules form a sandwich that comprises a β-hairpin (S6 and S7 for the nontoxic protein; S8 and S9 for PS2Aa) and an anti-parallel five-stranded β-sheet (S3, S9, S12, S5, and S8 for the nontoxic protein; S5, S6, S7, S10, S11, and S13 in PS2Aa) (Figure 1C–E) [28,29]. In PS2 family members, the β-hairpin forms a hydrophobic core with the inner surface of the β-sheet. This surface is covered with hydrophilic residues that surround the nucleus and are stabilized by hydrogen bonds. The arrangement of hydrophobic and hydrophilic residues is a critical factor involved in folding the protein [9]. In addition, three of the five strands of the β-sheet rearrange with the S1 strand to form a four-stranded β-sheet near the border of domain I, associated with the helices of domain I through hydrophobic interactions [28]. Site-directed mutagenesis was performed in the amphipathic β-strand of an epsilon toxin [9]. These mutations altered the characteristics of the channel formed in the lipid bilayer, suggesting that domain II is involved in the insertion and formation of lytic pores [9].

**Figure 2:**
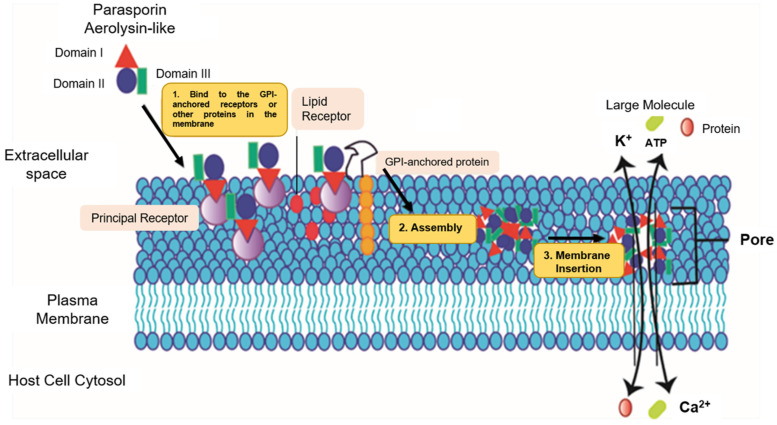
Action mode of aerolysin-like parasporins (PS2Aa). Figure adapted from [9]. According to this model, the mechanism of action could be as follows: 1. The solubilized protein binds to the GPI-anchored receptors at the N-terminus. 2. After C-terminal proteolytic digestion, the activated protein monomers assemble (oligomerization). 3. Through reorganization, a transmembrane β-barrel is formed.

Domain III is also involved in pore formation and has a loop that crosses the membrane [30]. It plays a vital role in the interaction between the individual monomers in the oligomer. In PS2, the five-stranded β-sheet is rearranged into a three-stranded β-sheet and two anti-parallel strands (S6, S11, S13/S14, S7, and S10) (Figure 1). These two β-sheets form a β-sandwich, similar to the β-sandwich in domain II [28]. The β-sheet structure is quite similar to the structure of the *Bt* A1470 nontoxic protein, which ends in two β-sheets: one with three strands and the other one with two (S4, S9, and S12; S5 and S8) (Figure 1) [29]. In PS2Aa1, some hydrophobic residues within the β-sandwich are exposed on both sides of the domain, forming small hydrophobic surface patches along the β-strands. A pair of anti-parallel β-strands (S4–S8 and S9–S11) (Figure 2) returns to the distal end of the domain, forming two loops [28]. The C-terminal residue is next to that pair of loops, pointing to its outer terminus. This residue is removed during proteolytic digestion, being a part of the hydrophobic nucleus inside the β-sandwich exposed to the solvent, creating a hydrophobic zone in the β-strand. These findings demonstrate the critical role of the C-terminal residue in oligomerization [9].

Sequence analysis using position-specific iterative (PSI)-BLAST indicated that the sequences of PS2, PS4, and PS5 are homologous to those of the β-PFT family [23,25,31], but they show different cytotoxic effects on normal cells [18]. Whereas PS4 did not show cytotoxic activity for any of the normal cell lines investigated [32], PS5 showed moderate cytotoxicity against the normal cell lines MRC-5 and UtSMC [18].

The high-molecular-weight PSs (Figure 1A) are more closely related to the insecticidal Cry proteins than the lower-molecular-weight PSs (Figure 1B). The bulk of the experimental data published to date related to high-molecular-weight PSs, and data from the determination of *Bt* have been a powerful tool for elucidating its role as a bio-controller of insect pests and disease vectors [33]. Therefore, it is imperative to consolidate the current information to characterize the great importance of the lower-molecular-weight PSs of the aerolysin type in medicine and biotechnology.

## 3. Effects of Parasporins on Cancer Cells

The mechanism of action of pore-forming proteins (PFPs) is dynamic, with three main steps: (1) the formation of water solubility, (2) self-assembly, and (3) insertion into the membrane, which leads to a pore suspected to be highly destructive for membrane integrity [34]. The points at which these proteins anchor to the membrane probably occur at specific receptors located in the microdomains rich in cholesterol and sphingolipids (lipid rafts), since these are requirements for GPI-anchored proteins, and the glucan region may be required for the binding and assembly in the membrane (Figure 2, part 1) [9]. Similarly, it was reported that the cell membrane receptor Beclin-1 could be important in the binding of three-domain parasporins (Parts 2 and 3, Figure 2) and that the Beclin-1 receptor is present in the mammary epithelium and epithelial carcinoma cells (Figure 2) [9,34,35].

The rearrangement of the domains typical for the classic protein model of three pore-forming domains does not occur for PS1 [36]. Therefore, its activity is not oriented to forming pores in the membrane [21,36]. PS1 was proposed to function as an activator of the apoptotic signaling pathway [14,19,37]. Selective cytotoxicity has been reported for the HeLa, Sawano, HepG2, HL-60, and MOLT-4 cell lines after PS1’s proteolytic activation by trypsin (Table 1) [6,38]. The activity of PS1 mainly involves modulating the influx of Ca^2+^ levels [6,21,39].

The PS2 mechanism of action likely starts with recognizing and binding to a receptor located in the cancer cells’ membranes [24], identifying lipid rafts, and anchoring the protein monomers in the periphery. The oligomers, resistant to sodium dodecyl sulfate (SDS), are embedded in the membrane, leading to its permeabilization [18,24]. Although PS2 is considered a selective pore-forming toxin, its primary receptors have not been fully elucidated [18].

Cells exposed to PS2 show morphological changes, including inflammation, blisters and lysis, microtubule disassembly, actin filament coiling, and fragmentation of the mitochondria and endoplasmic reticulum. PS2 resides in the plasma membrane and has been shown to activate apoptosis through caspases [14], triggering increased permeability [14,18,31]. These effects are induced by the accumulation of PS2 by large oligomers in the membrane’s lipid rafts [8,18,24,39]. In turn, association of PS2 with GPI is required for cytolytic action. By contrast, membrane cholesterol slightly affects the efficiency of oligomerization [1]. The activation of PS2 induced by proteinase K [25] leads to the exposure of specific regions that bind to the receptor [18,25].

PS3 acts as a pore former in cancer cells, thereby increasing cellular permeability [12,40]. Although PS3 is structurally similar to the Cry proteins, containing the five conserved blocks that characterize Cry [7,40], the PS3 and Cry proteins are fundamentally different due to a castor domain [7,40,41], which is present in many unrelated proteins and is presumed to enhance/induce carbohydrate-binding capacity [40]. Similar to the above-described PS, the mechanisms of action of PS3 remain largely unknown. Krishnan et al. suggested that PS3 is most likely pore forming [16], which leads to an imbalance in ATP, increased cell size, and membrane damage [40,41]. Its cytotoxic activity was evident in the HL-60 and HepG2 cell lines [7,40,41], but it did not affect HeLa cells [41].

Studies on PS3, PS4, PS5, and PS6 are limited compared to those on PS1 and PS2, and many action modes remain undetermined. PS4 shows homology with both Cry and pore-forming β-type aerolysin. It has been reported to be cytotoxic to the Caco-2, Sawano, and MOLT cell lines [5,41]. Its structure mainly comprises β-sheet domains, and its pore-forming activity is not dependent on cholesterol [21,41]. Cells treated with this protein show an increase in size due to an increase in the cytoplasmic compartment and shrinkage of the nucleus, leading to the rupture of the cytoplasmic membrane and cell death [42]. PS5 and PS6 are the most recently discovered PS proteins. They have three domains, similar to PS1 and PS2, and presumably have pore-forming activity. They have been reported to show cytotoxic activity in liver and cervical cancer cell lines. However, there is no further information on their mechanisms of action [14].

## 4. Perspectives on the Improvement of *Bt* Parasporins as an Innovative Strategy for Controlling Cancer Cells

By deciphering structure–function relationships, proteins with improved properties, e.g., desired thermal activity, selectivity, specificity, or folding, can be designed [43]. For example, engineered proteins with various substitutions of amino acids are used in receptor- and channel-protein-binding studies [44]. Protein engineering is called the synthesis of proteins with enhanced functionality in vitro and in vivo due to altered physical, chemical, or biological properties through genomic and post-genomic strategies. Genetic improvement is closely linked to complementary computational methods, which aim to optimize the generation of mutant libraries by simulating the experimental conditions of directed mutagenesis techniques [45,46,47,48]. In addition, other computational methods are oriented toward predicting protein structures and designing models that allow the prediction of molecular interactions and pinpoint amino-acid residues or regions at crucial positions in natural and mutant proteins [43,49]. The computational technique most widely used for studying the possible interactions of *Bt* Cry toxins with insect receptors is molecular docking, followed by molecular dynamics, which has proven to help predict the stability of the interactions and analyze the molecular mechanisms of action.

Florez et al. [50] obtained five Cry11 variants by DNA shuffling and showed the toxic activity against *Aedes aegypti* and *Culex quinquefasciatus* for three of them. Molecular docking simulations were performed for these three variants, and the amino acids with possible interactions were identified. BenFarhat-Touzri et al. [51] cloned and sequenced the Cry1D-133 toxin and determined its toxicity against *S. littoralis* larvae. Molecular docking simulations were performed to explain the enhanced toxicity of this toxin and showed that the number of toxin–receptor interactions was higher than that of the interactions exhibited by the Cry1D toxin.

The use of computational techniques based on molecular dynamics has enabled researchers to study the mechanisms of action of Cry toxins. The study of molecular dynamics has provided novel insights into the oligomerization of Cry toxins at a molecular level. Sriwimol et al. simulated the Cry4Ba structure with a three-dimensional reconstructed map for trimeric protein states. For the first time, they showed the need for membrane-induced conformational changes in Cry4Ba toxin monomers to allow the molecular assembly of a pre-pore trimer, which can be inserted into the target membranes to generate a lytic pore [52].

Other molecular dynamic studies have been applied to investigate the residue interactions relevant to the toxicity of the *Bt* Cry toxin family. Pacheco et al. discovered the importance of salt-bridge formation between α-helix residues from adjacent monomers for the toxicity and oligomerization of the Cry1Ab and Cry5Ba toxins by molecular dynamics’ simulations [53]. They showed a critical role for the salt bridge between the E101 and R99 residues of Cry1Ab [54]. Site-directed mutagenesis experiments confirmed decreased oligomerization and toxicity potential for Cry1Ab-E101K and Cry1Ab-R99E mutants.

Interestingly, the R99–E101 salt bridge is not fully conserved in Cry proteins, with both or one of the residues being different in Cry5Ba. However, Pacheco et al. showed that additional salt bridges with similar structural functions could also be formed in these Cry proteins. In conclusion, the computational analysis highlighted the importance of salt-bridge formation between the α-3 helices of adjacent monomers for inducing/facilitating a conformational change crucial for Cry toxicity [53].

### Genetic Improvement of Cry Protein as a Model to Be Followed for Parasporins

*Bt* is an excellent candidate for producing both natural and genetically enhanced PS proteins [55]. Studies on *Bt* have been in progress for over 100 years since the discovery of *Bt* in Japan [56]. During this time, several studies have established associations between the crystal morphology, protein sequences, and molecular weight, and the specific effects against its insecticidal targets [57]. *Bt* continues to be of great scientific interest, and it is one of the most studied biotechnological alternatives of biological origin on Earth. By contrast, PS remains a scarcely explored option, as few researchers have awakened their curiosity in PS and anticancer pore-forming proteins.

Various approaches have been taken to modify *Bt* toxins’ binding specificity and affinity, with the ultimate goal of producing genetically modified toxins that target new pest species and counteract the resistance developed in the field. The alteration of the binding affinity and specificity of the *Bt* toxin can come from domain exchanges, site-directed mutagenesis, truncation, and the generation and subsequent visualization of parasporal proteins in phage libraries containing mutant toxins [55].

Although there are no reports on the genetic improvement of PS-like parasporal proteins, here we review the technology applied to other types of *Bt* crystal proteins, which can serve as a methodological and scientific basis for obtaining PS proteins with improved cytotoxic activity against cancer cells. Table 2 presents several examples of the directed evolution techniques that have been successful for *Bt* proteins and might also be used shortly for PS modification and studies.

Domain exchanges between related proteins might have an increased toxicity towards the target cells. For Cry proteins, several examples are presented in Table 2. Site-directed mutagenesis at specific Domains or Loops is an efficient technique for obtaining potent toxins with enhanced toxicity. An example is Cry19A, for which, by substitutions in Domain II loops 1 and 2, the toxicity increased up to 42,000 times, concerning the parental [64]. Other techniques, such as truncated toxins or filtering of promising candidates with increased receptor affinity through Phage-display libraries, are presented in Table 2. Genetic improvement of 3D-Cry toxins is reviewed extensively by Susana Vilchez [75].

PSs require extensive structural and functional studies, which will help to unravel their complex but elegant mechanism of action leading to the cytotoxic effects upon cancer cell lines. Studies addressing this knowledge gap will help to elucidate the toxic action of PSs and define the structure–function relationship. Acknowledging its potential as anticancer molecules, PSs’ site-directed evolution studies might focus as well on analyzing the cellular permeability and the potency of its toxicity to cells as well as the selectivity. In the long term, the generation of PS variants might allow new alternatives to address the threat of cancer to the well-being of humans and the burden on the health system. The current knowledge highlights the potential role that bacterial proteins might play in generating novel anticancer molecules [9].

## 5. Conclusions

The low-molecular-weight PS proteins, such as the pore-forming toxin PS-2Aa1, induce apoptosis in target cells and have cytotoxic activity against several human cancer cell lines. Remarkably, low-molecular-weight PS proteins do not show detectable cytotoxic activity against normal cells in most cases [12]. Therefore, PS proteins have emerged as a viable, efficient, and natural alternative for combating cancer, one of the types of diseases with the highest mortality rate in humans. However, limited knowledge on these toxins at the molecular level, including the mechanism of action and the receptors targeted on cancer cells, is available. PS2 is a single protein with a proposed action mode based on its structure. Therefore, there is an urgent need to establish the structural properties and mechanism of action to scale in in vivo studies and take the next step toward developing valuable products for human health [75].

In addition, there is a need to identify and evaluate new *Bt* native strains with improved cytotoxic activity toward cancer cells and select candidates for further genetic improvement to obtain toxins with enhanced activity. PS proteins are in the process of being consolidated as a viable alternative for cancer treatment. Compared to other current approaches, they hold the potential to produce fewer side effects, improving both the treatment outcomes and quality of life of cancer patients.

## Figures and Tables

**Table 1:** Parasporin families, family-containing strains, molecular weights, target cells, cytotoxic activity, and references.

Parasporin	Strain (*Bt*)	Molecular Mass (kDa)	Target Cell Line	Cytotoxic Activity IC_50_ (μg/mL)	Ref.
PS1Aa1	A1190	81	MOLT-4 HL-60 HepG2 HeLa Jurkat Sawano Caco-2 A549	2.2 0.32 3.0 0.12 >10 >10 >10 >10	[9,13,14]
PS2Aa1	A1547	37	MOLT-4 Jurkat HL-60 HepG2 Sawano Caco-2 HCT116 CCRF-CEM	0.022 0.018 0.019 0.019 0.0017 0.013 10 27	[8,9,14,15]
PS3Aa1	A1462	93	MOLT-4 Jurkat HL-60 HepG2 HeLa Sawano	>10 >10 1.32 2.8 >10 >10	[9,14,16]
PS4Aa1	A1470	34	MOLT-4 HL-60 HepG2 Sawano TCS Caco-2	0.472 0.725 1.90 0.245 0.719 0.124	[9,14,17]
PS5Aa1	A1100	31	MOLT-4 Caco-2 HepG2 TCS HeLa Sawano.	0.075 0.30 0.049 0.046 0.08 0.065	[14,18]
PS6Aa1	M109/CP84	73	HepG2 HeLa Caco-2	2.3 7.2 >10	[14,19]

**Table 2:** Modifications made to *Bt* toxins to improve their efficacy.

Type of Modification	*Bt* Toxin	Target Insect	Increase or Decrease in Toxicity	Reference
Domain exchanges				
Domain III Exchange For Domain III of Cry1Ab.	mCry3Aa	*Diabrotica virgifera*	The toxicity increased ≥19%.	[58]
Domain III, II, I Exchange For Domains of Cry1Ac.	Cry9Aa	*Helicoverpa armigera*	The toxicity increased between 4.9 and 5.1 times, concerning parentals.	[59]
Domain III Exchange For Domain III of Cry1Ca.	Cry1Ab; Cry1Ac; Cry1Ba; Cry1Ea; Cry1Fa	*Spodoptera exigua*	Increased up to 5.5 times for Cry1Fa.	[60]
Domain III Exchange For Domain III of Cry1CAc	Cry1Ca; Cry1Fb; Cry1Ba; Cry1Da; Cry1Ea	*Heliothis virescens*	The toxicity increased 172 and 69.6 times more for Cry1Ca and Cry1Fb, respectively.	[61]
Domain exchanges of Domains II and III, between Cry1Ia and Cry1Ba.	Cry1Ia; Cry1Ba	*Leptinotarsa decemlineata*	The toxicity increased up to 1127 and 4.2 times, compared to Cry1Ba and Cry1Ia, correspondingly.	[62]
Site-directed mutagenesis				
Loops 1, 2, and 3, domain II substitution.	Cry4Ba	*Culex pipiens; Culex quinquefasciatus*	The toxicity increased up to 700 times.	[63]
Loops 1 and 2 domain II substitution	Cry19Aa	*Aedes aegypti*	The toxicity increased up to 42,000 times, concerning the parental.	[64]
Substitution in the domain II	Cry2Ab	*Anopheles gambiae*	The toxicity increased up to 6.75 times.	[65]
Loops 1 and 2 domain II substitution and deletions.	Cry1Aa	*Culex pipiens*	Change in insect target.	[66]
Substitution in the domain III	Cry1Ab	*Spodoptera frugiperda*	The toxicity increased up to 44 times, correspondingly to the parental.	[67]
Truncated toxins				
Truncation and selection of mutants, derived from a phage library	Cry1Ia	*Telchin licus*	The toxicity increased, showing mortality of 50% for approach.	[68]
Helix α-1 domain I truncation.	Cry1A	*Pectinophora gossypiella*	The toxicity increased up to 100 and 150 times for Cry1Ab and CryAc, respectively.	[69]
Helix α-1 domain I truncation.	Cry1A	*Plutella xylostella; Ostrinia nubilalis*	The toxicity increased ≥350 times, against resistant insects.	[70]
C-terminal truncation	Cry1C	*Spodoptera exigua*	The toxicity increased up to 4 times.	[71]
Phage-display library				
Selection of mutant toxins from a phage-display library based on their potential of binding.	Cry1Aa	*Bombyx mori*	Increased the receptor affinity potential up to 16 and 50 times more, contrasting the parentals.	[72]
Selection of mutant toxins from a phage-display library based on their potential of binding.	Cry8Ka	*Anthonomus grandis*	Increased the toxicity up to 3.2 times, contrasting the parental.	[73]
Selection of mutant toxins from a phage-display library based on their potential of binding in the domain II.	Cry1Aa	*Nilaparvata lugens*	The toxicity increased between 1.4 and 8.9 times, concerning parentals.	[74]

## Data Availability

Not applicable.
